# Exploring psychosocial factors influencing sexually transmitted infection intention testing among medical students: a cross-sectional study in two universities

**DOI:** 10.3389/fpubh.2024.1407070

**Published:** 2024-09-20

**Authors:** Valentina Loaiza-Guevara, María Alejandra Gómez Acosta, Angie Valeria Aguirre Álvarez, Valentina Agudelo Martínez, María Camila Montes Montoya, Alexandra Agudelo Ramírez, Juan S. Izquierdo-Condoy

**Affiliations:** ^1^Facultad de Medicina, Fundación Universitaria Autónoma de las Américas, Pereira, Colombia; ^2^Grupo de investigación Biomedicina, Fundación Universitaria Autónoma de las Américas, Pereira, Colombia; ^3^Grupo de Investigación en Salud y Comunidad (GISCO), Fundación Universitaria Autónoma de las Américas, Pereira, Colombia; ^4^One Health Research Group, Universidad de las Américas, Quito, Ecuador

**Keywords:** perceptions, previous experiences, psychosocial factors, sexually transmitted infections, medical students

## Abstract

**Background:**

Despite the significant global burden of sexually transmitted infections (STI), detection rates are poor. Acceptance of these tests is influenced by several factors that have not been explored among Colombian medical students.

**Objectives:**

The aim of this study was to describe the behaviors and psychosocial factors toward STI screening among medical students of two universities in Pereira, Colombia, between March and June 2020.

**Methods:**

An observational, cross-sectional study was conducted with 284 medical students in the first 3 years of undergraduate at two universities. An online, self-administered survey was conducted between March 2020 and June 2020, using an instrument from the “STI Testing Questionnaire,” to assess behaviors and psychosocial factors toward STI testing. Frequencies and percentages were used for descriptive analysis. The association between characteristics and psychosocial factors with STI testing intention was obtained using a logistic regression model. A *p*-value <0.05 was accepted as statistically significant for all analyses.

**Results:**

A total of 284 medical students participated in this study. The majority were female (56.7%), and 53.2% were from private universities. Eighty-four point 5% (84.5%) had risky sexual behaviors, and only 32.4% reported intentions to be tested for STIs. Among the psychosocial factors, 64.1% reported high social pressure, and 43.0% reported social fear. An association with the intention to undergo STI testing was identified in those who had been previously tested (OR = 2.486; 95% CI: 1.492–4.142) and in those who engaged in risky sexual behaviors (OR = 3.537; 95% CI: 1.437–8.704).

**Conclusion:**

Medical students exhibit a high prevalence of risky sexual behaviors but show a disturbing lack of intention to undergo STI screening. Prior experiences significantly influence screening intentions, while social pressure and fear also play a role. These insights can serve as a basis for targeted interventions to improve STI screening rates and enhance sexual health education among Colombian medical students.

## 1 Introduction

Sexually Transmitted Infections (STIs) represent a significant public health issue due to their high transmissibility, continuous and exponential growth, consequences in terms of morbidity and mortality, costs associated with the health system, and short-, medium-, and long-term complications (1). According to the World Health Organization (WHO), more than 1 million people between the ages of 15 and 49 contract an STI every day, most of them asymptomatic. Projections for 2020 indicated 374 million new infections from one of the four treatable STIs (chlamydia, gonorrhea, syphilis, and trichomoniasis) (2). Recent data indicates that HIV, viral hepatitis, and STIs cause 2.5 million deaths each year, with deaths from hepatitis-related causes increasing to 1.3 million in 2022, compared to 1.1 million in 2019. Increased notifications of STI cases are occurring in many WHO regions, which, together with the decrease in new HIV infections and viral hepatitis, indicate that STI control is not advancing fast enough to meet the Sustainable Development Goals for 2030 (3).

During the COVID-19 pandemic in 2020, there was a reported reduction in medical consultations and STI testing in countries like Spain due to lockdowns and social isolation measures (4). However, after the restrictions were lifted, there was a significant rebound in STIs in 2021, a phenomenon reported in various sources (4, 5). The greatest burden of STIs globally is found among adults aged from 15 to 49 years, including university students, who tend to underestimate the risk of contracting STIs and engage in high-risk behaviors such as inadequate condom use (2, 6, 7).

In Colombia, according to the National Institute of Health (INS), HIV has shown an increasing trend year after year, reaching 26.6 cases per 100,000 inhabitants in 2019 (8). The National Demographic and Health Survey showed that only three out of 10 young people have sufficient information about STIs (9). Additionally, the department of Risaralda in Colombia has the third-highest HIV incidence rate (40.9 cases per 100,000 inhabitants), with the infection affecting mostly the population of the municipality of Pereira between 20 and 34 years of age (10).

Screening tests are one of the pillars of STI management and control, bringing benefits for both individual and collective health by ensuring timely access to appropriate treatments and encouraging safer sexual behaviors (6). Cultural influences play a significant role in the area of secondary prevention of STIs. Cultural norms and values can affect attitudes toward sexual health and willingness to be screened. In many cultures, the stigma associated with STIs can be a major barrier to early detection and treatment (11).

Studies on STIs have shown that screening rates can be influenced by factors such as age, gender, ethnicity, education level, knowledge level, and systemic factors (8). Social and psychological factors such as perceptions of susceptibility and severity have also been shown to be predictors of STI testing behavior (12, 13). Research on behavioral theories has demonstrated their potential effect on various sexual behaviors, including the theory of planned behavior, the health belief model (HBM), and social cognitive theory (SCT) regarding barriers to STI testing (13). In this context, research has shown that self-esteem, shame, peer pressure, and lack of knowledge act as barriers that delay or impede the willingness to undergo testing for timely detection (14). In Colombia, stigma and discrimination in the workplace, family, and health services have been identified as the main barriers to accessing STI testing (15).

It is crucial to focus on the college student population and medical students. University students, due to their youth and high-risk behaviors, are a vulnerable group to STIs. Additionally, medical students, being in training to become health professionals, have a dual responsibility: managing their own health and promoting healthy practices in their future professional practice (16). However, the possible psychosocial factors associated with STI screening tests in undergraduate medical students in Colombia have not been evaluated so far. Therefore, the objective of this research was to identify and describe the demographic, academic, sexual behavior, and psychosocial factors associated with the intention to undergo STI screening tests in medical students from two universities in Pereira, Colombia.

## 2 Methods

### 2.1 Study design

We conducted a cross-sectional observational study through a self-administered online survey.

#### 2.1.1 Setting, population, and sample

Pereira is a Colombian municipality, the capital of the department of Risaralda located in the Eje Cafetero region. Online data were collected from students officially enrolled between the first and sixth semester in medical schools of two universities (one public and one private) located in the municipality of Pereira, between March and June 2020.

A non-probabilistic convenience sampling was used for the data collection process. Participants between 18 and 25 years of age, of both sexes, with a history of an active sexual life (having had at least one sexual relationship prior to filling out the questionnaire), who agreed to voluntarily participate in the survey through an electronic informed consent form were included in the research. Responses from participants outside the age ranges, with no sexually active life, or who did not accept the electronic informed consent were not included.

### 2.2 Study size

The required sample size was calculated using the following equation, designed to calculate samples in infinite or unknown populations (17):


$$n=\frac{Z^{2}.\left(p.q\right)}{e^{2}}$$

Starting with a confidence level of 95% (*Z* = 95%), a margin of error of 6% (*e* = 6%), and an expected positive (*p*) and negative (*q*) distribution of 50%, a minimum of *n* = 267 completed questionnaires were obtained.

### 2.3 Data measurement and questionnaire

A survey developed from the STI Testing Questionnaire, based on the theory of planned behavior (TPB), the HBM, and the SCT theories (18–21), and previously validated by Martin-Smith et al. with UK university students, was used, demonstrating a Cronbach’s alpha coefficient > 0.7 (22).

An online, self-reported, survey-type questionnaire, adapted from the STI Testing Questionnaire was utilized (22). The tool was validated through pilot testing among 21 participants from the medical schools of the two participating universities, followed by feedback that included the development of relevant corrections related to understanding terminology and wording. Once the errors were corrected, a structured and uniform questionnaire consisting of 91 questions was obtained. Additionally, the final version of the questionnaire demonstrated an internal consistency with a Cronbach’s alpha coefficient of 0.67. A version of the final questionnaire translated into English was included as a Supplementary file.

The online questionnaire consisted of eight sections: (1) Informed consent; (2) Sociodemographic data made up of 11 questions that assessed variables such as sex, age, year of study, type of university, religion, sexual risk behaviors, and intention to be tested for STIs; (3) Knowledge about STIs made up of 17 questions; (4) Social pressure for STI testing made up of 8 questions; (5) Direct attitudes toward STI testing comprised of 8 questions; (6) Indirect attitudes toward STI testing comprised of 21 questions; (7) Social fear toward STI testing comprised of 8 questions; (8) Self-efficacy with 17 questions related to medical students’ confidence toward STI testing.

### 2.4 Variables

The type of university was classified into public and private according to the university’s source of funding. To assess sexual behavior, sexual risk behaviors were measured based on four questions about sexual activity without condom use, with casual or regular partners. If at least one of the questions indicated a risk behavior, the participant was categorized as having sexual risk behavior. Additionally, the history of STI testing and the intention to undergo STI testing were explored.

To evaluate psychosocial factors, six variables (indicators) were studied:

- Knowledge about STI testing, defined as the information and understanding about STI transmission, symptoms, and prevention, was assessed through the evaluation of questions on STI transmission, symptoms, and prevention of STIs. To define the level of knowledge, a value of 1 point was assigned for each correct answer and 0 points for incorrect answers. This resulted in a maximum score of 17 points and a minimum score of 0 points (participants who answered all questions incorrectly). Thus, three categories of STI knowledge level were defined: high knowledge level (scores between 12 and 17 correct answers), medium knowledge level (scores between 7 and 11 correct answers), and low knowledge level (for scores between 6 and 0 correct answers).
- Social pressure, defined as the influence exerted by society or peers to conform to certain behaviors or attitudes regarding STI testing, was assessed subjective norms, i.e., perceived pressure to perform or not to perform an STI test in relation to four groups: partners, friends, family, and health professionals. For each question, the belief score was based on a 5-category Likert-type scale (completely disagree, somewhat disagree, neither disagree nor agree, somewhat agree, and completely agree) through which an overall score between −40 and + 40 was obtained and classified into three categories, where −40 to −13 represent low perceptions of social pressure to have an STI test, from −13 to 13 moderate perceptions of social pressure, and between 13 and 40 for a high perception of social pressure.
- Attitudes toward STI testing were measured through two approaches including direct attitudes and indirect attitudes. Direct attitudes, defined as explicit expressions of approval or disapproval toward a behavior, were evaluated the participants’ stance by means of a Likert-type scale of 5 categories (completely disagree, somewhat disagree, neither disagree nor agree, somewhat agree, and completely agree) through a possible score between 8 and 35, finding three possible categories: positive direct attitude (between 35 and 27), moderate direct attitude (between 17 and 26), and negative direct attitude (between 8 and 16). Indirect attitudes defined as subtle or implied expressions of approval or disapproval, were measured using a Likert-type scale with five categories (completely disagree, somewhat disagree, neither disagree nor agree, somewhat agree, and completely agree). The scores of the questions were summed to obtain a maximum score of 55 and a minimum of 11, measuring the indirect attitude in three categories: positive perceptions (between 55 and 41), neutral perceptions (between 40 and 26), and negative perceptions (between 25 and 11).
- Social fear, defined as the apprehension of negative consequences or stigma from others impacting decisions like STI testing, was explored using a 5-category Likert-type scale (completely disagree, somewhat disagree, neither disagree nor agree, somewhat agree, and completely agree) toward situations you would face with STI testing. The question scores were summed to create a composite score ranging from 8 to 40, with low perceived social fear ranging from 8 to 19, moderate perceived fear toward STI testing ranging from 20 to 29, and high perceived social fear with scores ranging from 30 to 40.
- Self-efficacy, defined as the belief in one’s ability to successfully perform a behavior, was measured using a 5-category Likert-type scale questions of confidence (0% confidence, 25% confidence, 50% confidence, 75% confidence, 100% confidence). Item scores were summed to create a composite score ranging from a minimum of 12 to a maximum of 60. From this, self-efficacy was organized into three categories, where a score between 12 and 27 represents low self-efficacy to undergo STI testing, between 28 and 44 represents moderate self-efficacy, and high self-efficacy between 45 and 60.

### 2.5 Data collection and management

The online platform Google Forms facilitated data collection. Participants accessed the survey through a unique link disseminated on social networks, including “Facebook” and “WhatsApp.” The survey link was distributed by the research team in the official Facebook and WhatsApp groups for medical students at the participating universities. Once participants entered the questionnaire, the survey preamble briefly described the objectives of the study, promised confidentiality, and requested informed consent. Participants had to accept the Participation Agreement to access the survey through an electronic informed consent question, reinforcing anonymity.

To maintain a high degree of data integrity, the researchers meticulously examined all responses for possible errors or inconsistencies, such as respondents choosing all available responses or duplicate responses and excluded these questionnaires from the final sample. Initially, we had 325 survey responses. After rigorous filtering, 41 responses were excluded mainly due to systematic errors, such as participants completing the questionnaire despite indicating they had not had sexual intercourse, surveys with inconsistent age ranges, or surveys that only contained demographic data. A total of 284 valid responses were obtained and included in the study.

### 2.6 Bias

To control for possible biases during data collection and management, we implemented several strategies. To avoid duplicate submissions, the Google Forms platform was configured to limit one response per IP device. In addition, our questionnaire design was intentionally selected to avoid collecting identifiable information such as IP addresses. To further minimize bias during the analysis phase, each member of the research team reviewed the results independently. When discrepancies arose, they were collaboratively resolved to ensure that only valid and genuine responses were incorporated into our final findings. These measures significantly strengthened the reliability and credibility of our research results.

### 2.7 Statistical analysis

Our study used descriptive statistical measures to analyze the responses to each categorical item of the questionnaire, including the calculation of frequencies and percentages. In addition, a logistic regression model was used to estimate the association between participant characteristics, knowledge, and psychosocial factors with the intention to screen for STIs, expressed by Odds Ratio (OR) and 95% confidence intervals (95% CI). The tests were two-tailed, and we considered statistical significance with *p* values less than 0.05. For both descriptive and inferential analyses, we used IBM SPSS Statistics for Windows, version 26.0 (IBM Company, Chicago, IL, United States).

## 3 Results

### 3.1 Demographic characteristics

A total of 284 medical students were included, the majority being 56.7% (*n* = 161) female, with 70.1% (*n* = 199) aged 20 years or younger, and 79.2% (*n* = 225) considering themselves believers in some religion. Additionally, slightly more than half (53.2%) reported studying at a private university, and the majority of participants were in their second year of medical school (40.8%; Table 1).

**Table 1:** Demographic characteristics and sexual behaviors of medical students.

		*n*	%
**Demographic characteristics**			
Sex	Female	161	56.7
	Male	123	43.3
Age (years)	≤ 20	199	70.1
	> 20	85	29.9
Univesity type	Private	151	53.2
	Public	133	46.8
Year of study	First	108	38.0
	Second	116	40.8
	Third	60	21.1
Religion	Believer	225	79.2
	Nonbeliever	59	20.8
**Sexual behaviors**			
STI pre-screening	No	151	53.2
	Yes	133	46.8
Intention to screen for STIs	No	192	67.6
	Yes	92	32.4
Sexual risk behaviors	No	44	15.5
	Yes	240	84.5

Regarding sexual behaviors, 53.2% (*n* = 151) of the participants reported not having been previously tested for STIs, and only 32.4% (*n* = 92) reported an intention to undergo testing for asymptomatic STIs (Table 1). However, 84.5% (*n* = 240) were identified as having risk sexual behaviors. Within this group, the most prevalent risk behavior was having ever had vaginal intercourse without condom use with a regular partner, reported by 91.3% (*n* = 219), followed by having ever had vaginal intercourse without condom use with an occasional partner, reported by 35% (*n* = 84).

### 3.2 Psychosocial factors

#### 3.2.1 Knowledge

Regarding the knowledge of the participants with STI testing, it was evident that the features with the greatest lack of knowledge, defined by the percentage of incorrect answers, were related to the diagnosis of diseases such as chlamydia (65.8%), gonorrhea (70.4%), and syphilis (59.9%). The questions with the highest knowledge were related to the probability of infection if a condom is not used (92.6% correct answers), symptoms of STIs (89.8%), and treatment of some STIs (82.7%; Supplementary Table 1). However, the overall assessment of knowledge showed that 76.1% (*n* = 216) of participants had a high level of knowledge (Table 2).

**Table 2:** Characteristics of psychosocial factors toward STI screening in medical student.

Psychosocial factors		*n*	%
Knowledge	Low	6	2.1
	Moderate	62	21.8
	High	216	76.1
Social pressure	Low	10	3.5
	Moderate	92	32.4
	High	182	64.1
Direct attitudes	Positive	284	100.0
Indirect attitudes	Negative	2	0.7
	Neutral	227	79.9
	Positive	55	19.4
Social fear	Low	37	13.0
	Moderate	125	44.0
	High	122	43.0
Self-efficacy	Low	1	0.4
	Moderate	46	16.2
	High	237	83.5

#### 3.2.2 Social pressure

The evaluation of social pressure toward STI testing showed that participants were influenced by the pressure exerted by others, particularly sexual partners (48.2%), family members (35.2%), and doctors and health professionals (55.6%; Supplementary Table 2). In this context, the overall assessment of the influence that social pressure can exert on the development of STI screening tests found a high degree of social pressure in 64.1% (*n* = 182) of the participants (Table 2).

#### 3.2.3 Direct attitudes

Direct attitudes toward STI screening tests showed a predominant stance in favor, as the majority of students disagreed with negative characteristics of screening tests, such as considering them a waste of time, painful, harmful, or being unimportant (Supplementary Table 3). Consequently, the overall estimate of direct attitudes toward STI testing revealed a completely positive stance in 100.0% (*n* = 284) of participants (Table 2).

#### 3.2.4 Indirect attitudes

Indirect attitudes, on the other hand, showed that the aspects most likely to influence a negative stance against STI screening tests included viewing them as something shameful (37.0%), believing they could affect the future and relationships (56.7%), or considering them detrimental to career prospects (59.2%; Supplementary Table 4). Overall, indirect attitudes were defined by a neutral or indifferent stance toward STI screening tests in 79.9% (*n* = 227) of the participants (Table 2).

#### 3.2.5 Social fear

Regarding social fear, the main factors in the screening tests that could lead to social fear were concern about sexual partner reaction (50.5%), concern about parental reaction (40.0%), and feeling puzzled (35.9%; Supplementary Table 5). In this context, social fear toward STI testing and its results showed a significant influence on students, with 44.0% (*n* = 125) having a moderate level of social fear and 43.0% (*n* = 122) experiencing a high level of social fear (Table 2).

#### 3.2.6 Self-efficacy

The self-efficacy events that generated the greatest insecurity in the participants were that they considered that they would be the greatest in the waiting room (20.7%) and that the result would be made public (42.3%), while among the factors to which they seemed to attach no importance were related to the time available for testing, such as weekends or in the evenings (Supplementary Table 6). Thus, a high self-efficacy effect is evident in 83.5% (*n* = 237) of students (Table 2).

### 3.3 Factors associated with the intention to be tested for STIs

Within the demographic characteristics of the respondents, only those related to education showed effects on the intention to be tested for STIs, showing that participants from public universities would be less inclined to be screened (OR = 0.418; 95% CI 0.248–0.703). While participants in the second year of undergraduate medical school would be more inclined (OR = 1.790; 95% CI 1.017–3.150; Table 3).

**Table 3:** Association between demographic and sexual behavior characteristics and the intention to undergo STI screening in medical students.

		Intention to undergo STI screening				
		No (*n* = 192)		Yes (*n* = 92)		OR (CI 95%)
		*n*	%	*n*	%	
Sex	Female *ref.*	113	70.2	48	29.8	
	Male	79	64.2	44	35.8	1.311 (0.795–2.161)
Age (years)	≤ 20 *ref.*	141	70.9	58	29.1	
	> 20	51	60.0	34	40.0	1.621 (0.953–2.756)
University type	Private *ref.*	89	58.9	62	41.1	
	Public	103	77.4	30	22.6	**0.418 (0.248–0.703)**
Year of study	First *ref.*	79	73.1	29	26.9	
	Second	70	60.3	46	39.7	**1.790 (1.017–3.150)**
	Third	43	71.7	17	28.3	1.077 (0.532–2.178)
Religion	Nonbeliever *ref.*	41	69.5	18	30.5	
	Believer	151	67.1	74	32.9	1.116 (0.600–2.075)
STI pre-screening	No *ref.*	116	76.8	35	23.2	
	Yes	76	57.1	57	42.9	**2.486 (1.492–4.142)**
Sexual risk behaviors	No *ref.*	38	86.4	6	13.6	
	Yes	154	64.2	86	35.8	**3.537 (1.437–8.704)**

Statistically significant associations are marked in bold.

For their part, sexual behaviors exhibited a significant association with intent to be screened, as there was an association toward positive intent in those who had been previously screened for STIs (OR = 2.486; 95% CI 1.492–4.142) and in those with risky sexual behaviors (OR = 3.537; 95% CI 1.437–8.704; Table 3).

Most of the psychosocial factors did not show significant effects on the participants’ intention to be tested for STIs. However, within the indirect attitudes, apparently having positive direct attitudes was associated with the intention to be screened (OR = 2.187; 95% CI 1.198–3.992; Table 4).

**Table 4:** Association between psychosocial factors and intention to undergo STI screening among medical students.

		Intention to undergo STI screening				
		No (*n* = 192)		Yes (*n* = 92)		OR (CI 95%)
Psychosocial factors		*n*	%	*n*	%	
Knowledge	Low *ref.*	5	83.3	1	16.7	
	Moderate	46	74.2	16	25.8	1.739 (0.188–16.031)
	High	141	65.3	75	34.7	2.659 (0.305–23.183)
Social pressure	Low *ref.*	9	90.0	1	10.0	
	Moderate	72	78.3	20	21.7	2.500 (0.298–20.923)
	High	111	61.0	71	39.0	5.756 (0.713–46.421)
Indirect attitudes	Neutral *ref.*	161	70.9	66	29.1	
	Negative	2	100.0	0	0.0	0.485 (0.023–10.254)
	Positive	29	52.7	26	47.3	**2.187 (1.198–3.992)**
Social fear	Low *ref.*	23	62.2	14	37.8	
	Moderate	92	73.6	33	26.4	0.589 (0.271–1.278)
	High	77	63.1	45	36.9	0.960 (0.449–2.051)
Self-efficacy	Low *ref.*	1	100.0	0	0.0	
	Moderate	37	80.4	9	19.6	0.760 (0.028–20.175)
	High	154	65.0	83	35.0	1.621 (0.065–40.244)

Statistically significant associations are marked in bold.

## 4 Discussion

This study evaluated psychosocial factors associated with intention to undergo STI screening in medical students. To our knowledge, this is the first exploration in medical students in South America. Using an online questionnaire, we gathered data from a sample of 284 sexually active medical students, predominantly women, with a significant inclination toward religious beliefs. However, neither gender nor religious beliefs showed significant associations with the intention to get tested for STIs. Previous research has highlighted the effect of gender on testing for different STIs, often finding differences in favor of women and men in various infections (23–25). This difference is mostly attributed to health promotion efforts focused on women, especially in cases of diseases such as chlamydia and gonorrhea (26, 27). In our sample, however, we believe that both men and women are equally exposed to academic information about STIs, which may mitigate the effect of gender on the intention to test for STIs.

Academic variables showed an influence on participants’ intention to undergo STI testing. For example, studying at a public university was associated with a lower intention to undergo testing. This can be explained by several factors, including the socioeconomic disparities to which students at public universities in Colombia are exposed (28), which may result in financial barriers that affect access to medical consultations and STI testing (29). Additionally, our findings showed an association between students who have progressed further in their year of study and their intention to undergo STI testing. Although this was not statistically significant among third-year students, it shows a trend similar to those observed in other studies on college students (22). In this context, this inclination could be attributed to increased exposure to sexual health and STI-related topics in the curriculum, which raises awareness of STI risks and consequences, thereby increasing student’s intention to undergo testing (14).

Regarding sexual behavior, 84.5% of medical students reported engaging in risky sexual behaviors, such as having multiple sexual partners and unprotected intercourse. This rate is comparable to findings among university students in 2019 in Barranquilla, Colombia (87.0%) (30), and Polish university students (87%) (31), but significantly higher than rates reported among Ethiopian university students (44.0%), female medical students in Cuba (74.5%), and Nigerian medical students (6.1%) (32–34). Despite the high prevalence of risky behaviors, the proportion of students who had undergone previous STI screening or intended to do so in the future was notably low. This discrepancy could hinder infection detection and increase the likelihood of transmission and complications (27). However, in this context, curiously, we found that previous STI screening and risky sexual behaviors were the factors most associated with the intention to undergo STI screening (OR = 2.486; 95%CI 1.492–4.142, OR = 3.537; 95%CI 1.437–8.704, respectively). These findings somewhat contrast with the HBM, which suggests that individuals, based on their perceived vulnerability or susceptibility, take certain actions (35). In this scenario, those who perceive themselves to have a lower risk of contracting a disease are more likely to engage in riskier behaviors, including not intending to undergo screening tests. We believe this contradiction could be significantly influenced by the environment—specifically, university medical students who have a high level of knowledge about STIs. Despite understanding the potential consequences of their actions, they may still engage in risky sexual behaviors and be more willing to undergo screening tests.

On the other hand, psychosocial factors have been shown to have important effects on the intention to be tested for STIs in college students (22). Although most of the psychosocial factors did not show a statistically significant association with the intention to be tested for STIs in our research, the evaluation of psychosocial factors showed important details. For example, in most of the sample, the effects of the opinion of individuals in the close environment such as friends or family members marked an important effect on the social pressure that medical students may feel about STI testing. This is complemented by evidence that the reaction of parents and partners mainly makes up the social fear that participants may feel about STI testing and its outcome. In this regard, previous research has found that increased social pressure significantly affects intention and behavior toward early testing (6), as well as that fear, stigma, and clinical environment were critical to whether people would participate in screening services (36). This is explained in light of TPB theory, which posits that social pressure (subjective norms, behavioral control) is a modulator of people’s behavior (37).

The levels of knowledge about STIs among participants were encouraging, as only 21.8% demonstrated moderate knowledge and only 2.1% showed low knowledge about the transmission, symptoms, and prevention of STIs. These findings align with previous studies that identified high levels of knowledge about STIs in medical students in other regions of Colombia (38, 39). Although the level of knowledge was predominantly high and did not have a statistically significant effect on the intention to undergo STI screening among students, significant error rates were found related to the diagnosis of different STIs. These findings highlight the need to improve the knowledge of medical students in these areas, especially in the diagnosis of STIs.

Regarding the participants’ attitudes, although direct attitudes appeared to be completely positive, aspects within the participants’ indirect attitudes influence to stop medical students from getting tested for STIs, especially related to the possible repercussion of the result on their future life, evidencing a complex influence on the factors that revolve around STI screening. This idea is reinforced given that positive indirect attitudes demonstrated a positive association (OR = 2.187; 95%CI 1.198–3.992) toward getting tested for STIs, and this is consistent with research by Shepherd et al., in which it was evident that having an unfavorable attitude toward visiting a clinic negatively predicted screening (40), so such attitudes may deter young people from routine screening.

Although this research was conducted during the initial stage of the COVID-19 pandemic, we believe that the self-report design allowed us to explore the opinions of medical students without the quarantine affecting their views on STI screening tests. This is particularly relevant since data on risky sexual behaviors were collected for 1 year prior to data collection.

Furthermore, this research sheds light on a previously underexplored area concerning the detection of STIs among medical students in Colombia and South America. It reveals the challenges and factors that directly influence STI screening policies in sexually active and high-risk groups.

Regarding risky practices, we emphasize the importance of these findings in shaping health strategies within the Colombian and South American contexts. The goal is to include young populations, including university students, in strategies that promote the avoidance of risky sexual behaviors and highlight the importance and benefits of STI detection tests for personal health.

## 5 Limitations

This study had several limitations, including selection bias due to the use of convenience sampling and self-administered questionnaires. This was primarily because of the COVID-19 health crisis, which made it impossible to collect responses in person. Consequently, the results of this research may exhibit selection bias, as students less concerned about STIs might have been less interested in responding to the survey. Although the distribution aimed to use the platforms most commonly used by students, the response rate could have been affected by excluding students with limited access to the Internet or the social networks used for questionnaire distribution. This limitation could impact the sample size and the representativeness of the results. Additionally, to prevent duplicate responses, the number of responses was restricted to one per device using the features of the Google Forms platform.

Moreover, explorations of phenomena associated with STIs, such as this one, are susceptible to social acceptability bias. This bias could arise from students fearing to answer truthfully due to concerns about the anonymity of their responses. In this context, the researchers made efforts to reassure students about strict anonymity, including the use of an anonymous questionnaire in which none of the responses included identifiable participant information such as names, email addresses, or telephone numbers. Additionally, the survey included a statement guaranteeing the confidentiality of the responses to be collected, so participants felt secure about their privacy. Furthermore, the acceptance of a mandatory informed consent by each participant emphasized that responses would be anonymous and confidential, and stressed the importance of honesty in completing the instrument.

## 6 Conclusion

Medical students from two universities in Pereira, Colombia exhibited a high prevalence of risky sexual behaviors. However, there is a concerning trend of low intention to undergo screening for STIs, putting this group at a higher risk of STI transmission and associated complications.

Previous experiences, including previous screening tests and engagement in risky sexual behaviors, showed a significant effect on the intention to undergo STI screening.

While many psychosocial factors did not show associations with STI testing intentions, the influence of environmental factors was evident through the impact of social pressure and social fear on STI screening behaviors.

The findings of this study provide valuable insights into the underlying factors and enable the development of targeted interventions aimed at addressing barriers to STI screening. These interventions can promote a more comprehensive approach to sexual health education and promotion within the medical student in Pereira, and other Colombian localities.

## Data Availability

The original contributions presented in the study are included in the article/Supplementary material, further inquiries can be directed to the corresponding author.
