# Supplementary material for: Exploring psychosocial factors influencing sexually transmitted infection intention testing among medical students: a cross-sectional study in two universities
Source: Front Public Health. 2024 Sep 20;12:1407070. doi: 10.3389/fpubh.2024.1407070 (PMC11451435; doi:10.3389/fpubh.2024.1407070)
Supplement: Supplementary file 1 [file Table_1.DOCX]

Supplementary Material

# Supplementary Table 1. Knowledge about Sexually Transmitted Infections in medical students.

|  |  | **n** | **%** |
| --- | --- | --- | --- |
| You can always tell if you have an STI, as you would have symptoms (e.g., changes in your body) | Correct | 204 | 71.8% |
|  | Incorrect | 80 | 28.2% |
| The symptoms of all sexually transmitted diseases are painful | Correct | 255 | 89.8% |
|  | Incorrect | 29 | 10.2% |
| If you only have unprotected sex once, you won't get infected with an STI | Correct | 263 | 92.6% |
|  | Incorrect | 21 | 7.4% |
| People who use condoms are always safe from all STIs | Correct | 227 | 79.9% |
|  | Incorrect | 57 | 20.1% |
| Some STIs can cause infertility in women and men | Correct | 174 | 61.3% |
|  | Incorrect | 110 | 38.7% |
| Not All Sexually Transmitted Infections Can Be Cured | Correct | 226 | 79.6% |
|  | Incorrect | 58 | 20.4% |
| Most STIs will go away on their own | Correct | 227 | 79.9% |
|  | Incorrect | 57 | 20.1% |
| Some STIs can be treated with antibiotics | Correct | 235 | 82.7% |
|  | Incorrect | 49 | 17.3% |
| You have to pay to get tested | Correct | 128 | 45.1% |
|  | Incorrect | 156 | 54.9% |
| If you don't have symptoms (but want a test), the best place to get tested is at your Health Care Provider (EPS) | Correct | 187 | 65.8% |
|  | Incorrect | 97 | 34.2% |
| Are aware of the costs of getting tested for STIs | Correct | 85 | 29.9% |
|  | Incorrect | 199 | 70.1% |
| Know where to get tested for STIs | Correct | 197 | 69.4% |
|  | Incorrect | 87 | 30.6% |
| Requires a medical prescription to be tested for one or more STIs | Correct | 156 | 54.9% |
|  | Incorrect | 128 | 45.1% |
| Chlamydia can be diagnosed by taking a sample of your urine | Correct | 97 | 34.2% |
|  | Incorrect | 187 | 65.8% |
| Gonorrhea can be diagnosed by taking a sample of your urine | Correct | 84 | 29.6% |
|  | Incorrect | 200 | 70.4% |
| HIV can be diagnosed by taking a sample of your urine | Correct | 152 | 53.5% |
|  | Incorrect | 132 | 46.5% |
| Syphilis can be diagnosed by taking a sample of your urine | Correct | 114 | 40.1% |
|  | Incorrect | 170 | 59.9% |

**Supplementary Table 2.** Questions of social pressure towards STI testing in medical students.

|  |  | **n** | **%** |
| --- | --- | --- | --- |
| My sexual partners would like to be tested for STIs | Strongly disagree | 40 | 14.1% |
|  | Somewhat disagree | 11 | 3.9% |
|  | Neither agree nor disagree | 65 | 22.9% |
|  | Somewhat agree | 31 | 10.9% |
|  | Strongly agree | 137 | 48.2% |
| My friends would want to be tested for STIs | Strongly disagree | 63 | 22.2% |
|  | Somewhat disagree | 28 | 9.9% |
|  | Neither agree nor disagree | 91 | 32.0% |
|  | Somewhat agree | 44 | 15.5% |
|  | Strongly agree | 58 | 20.4% |
| My family would like to be tested for STIs | Strongly disagree | 49 | 17.3% |
|  | Somewhat disagree | 17 | 6.0% |
|  | Neither agree nor disagree | 76 | 26.8% |
|  | Somewhat agree | 42 | 14.8% |
|  | Strongly agree | 100 | 35.2% |
| Doctors and health care professionals would want me tested for STIs | Strongly disagree | 37 | 13.0% |
|  | Somewhat disagree | 10 | 3.5% |
|  | Neither agree nor disagree | 39 | 13.7% |
|  | Somewhat agree | 40 | 14.1% |
|  | Strongly agree | 158 | 55.6% |
| If I knew my doctors and health care professionals wanted me to get tested for STIs, I would | I definitely wouldn't take the exam | 5 | 1 .8% |
|  | Indifferent | 8 | 2 .8% |
|  | Maybe I'd take the test | 50 | 17 .6% |
|  | I would definitely take the exam | 221 | 77 .8% |
| If I knew my sexual partners wanted me to get tested for STIs, I would | I definitely wouldn't take the exam | 6 | 2 .1% |
|  | Maybe I wouldn't take the exam | 1 | 0 .4% |
|  | Indifferent | 12 | 4 .2% |
|  | Maybe I'd take the test | 79 | 27 .8% |
|  | I would definitely take the exam | 186 | 65 .5% |
| If I knew my friends wanted me to get tested for STIs, I would | I definitely wouldn't take the exam | 19 | 6 .7% |
|  | Maybe I wouldn't take the exam | 28 | 9 .9% |
|  | Indifferent | 71 | 25 .0% |
|  | Maybe I'd take the test | 76 | 26 .8% |
|  | I would definitely take the exam | 90 | 31 .7% |
| If I knew my family wanted me to get tested for STIs, I would | I definitely wouldn't take the exam | 9 | 3 .2% |
|  | Maybe I wouldn't take the exam | 7 | 2 .5% |
|  | Indifferent | 29 | 10 .2% |
|  | Maybe I'd take the test | 92 | 32 .4% |
|  | I would definitely take the exam | 147 | 51 .8% |

**Supplementary Table 3.** Direct attitudes toward STI testing of medical students.

| **Getting tested for STIs is:** |  | **n** | **%** |
| --- | --- | --- | --- |
| A waste of time | Strongly disagree | 284 | 100.0% |
| Good for me | Strongly disagree | 284 | 100.0% |
| Painful | Completely agree | 16 | 5.6% |
|  | Strongly disagree | 268 | 94.4% |
| Boring | Completely agree | 16 | 5.6% |
|  | Strongly disagree | 268 | 94.4% |
| Imprudent | Strongly disagree | 284 | 100.0% |
| Prejudicial | Completely agree | 1 | 0.4% |
|  | Strongly disagree | 283 | 99.6% |
| Unattractive | Completely agree | 85 | 29.9% |
|  | Strongly disagree | 199 | 70.1% |
| Something unimportant | Completely agree | 1 | 0.4% |
|  | Strongly disagree | 283 | 99.6% |

**Supplementary Table 4.** Indirect attitudes toward STI testing in medical students.

| **If I were to be tested for STIs I would:** |  | **n** | **%** |
| --- | --- | --- | --- |
| I'm going to stay healthy | Strongly disagree | 6 | 2.1% |
|  | Somewhat disagree | 8 | 2.8% |
|  | Neither disagree, nor agree | 41 | 14.4% |
|  | Somewhat agree | 68 | 23.9% |
|  | Completely agree | 161 | 56.7% |
| I'll feel calm | Strongly disagree | 4 | 1.4% |
|  | Somewhat disagree | 6 | 2.1% |
|  | Neither disagree, nor agree | 15 | 5.3% |
|  | Somewhat agree | 52 | 18.3% |
|  | Completely agree | 207 | 72.9% |
| It will be embarrassing | Strongly disagree | 105 | 37.0% |
|  | Somewhat disagree | 51 | 18.0% |
|  | Neither disagree, nor agree | 60 | 21.1% |
|  | Somewhat agree | 53 | 18.7% |
|  | Completely agree | 15 | 5.3% |
| I'm going to get information and advice | Strongly disagree | 5 | 1.8% |
|  | Somewhat disagree | 2 | 0.7% |
|  | Neither disagree, nor agree | 10 | 3.5% |
|  | Somewhat agree | 52 | 18.3% |
|  | Completely agree | 215 | 75.7% |
| It's going to be stressful | Strongly disagree | 59 | 20.8% |
|  | Somewhat disagree | 47 | 16.5% |
|  | Neither disagree, nor agree | 80 | 28.2% |
|  | Somewhat agree | 69 | 24.3% |
|  | Completely agree | 29 | 10.2% |
| I'm going to get comfortable | Strongly disagree | 12 | 4.2% |
|  | Somewhat disagree | 32 | 11.3% |
|  | Neither disagree, nor agree | 110 | 38.7% |
|  | Somewhat agree | 66 | 23.2% |
|  | Completely agree | 64 | 22.5% |
| It will negatively affect my future and my relationships | Strongly disagree | 161 | 56.7% |
|  | Somewhat disagree | 46 | 16.2% |
|  | Neither disagree, nor agree | 51 | 18.0% |
|  | Somewhat agree | 11 | 3.9% |
|  | Completely agree | 15 | 5.3% |
| I'll feel responsible | Strongly disagree | 6 | 2.1% |
|  | Somewhat disagree | 1 | 0.4% |
|  | Neither disagree, nor agree | 13 | 4.6% |
|  | Somewhat agree | 57 | 20.1% |
|  | Completely agree | 207 | 72.9% |
| It's going to take me a long time | Strongly disagree | 91 | 32.0% |
|  | Somewhat disagree | 63 | 22.2% |
|  | Neither disagree, nor agree | 95 | 33.5% |
|  | Somewhat agree | 26 | 9.2% |
|  | Completely agree | 9 | 3.2% |
| I will get the treatment I need before I have complications | Strongly disagree | 4 | 1.4% |
|  | Somewhat disagree | 4 | 1.4% |
|  | Neither disagree, nor agree | 27 | 9.5% |
|  | Somewhat agree | 64 | 22.5% |
|  | Completely agree | 185 | 65.1% |
| It will negatively affect my future and career prospects | Strongly disagree | 168 | 59.2% |
|  | Somewhat disagree | 46 | 16.2% |
|  | Neither disagree, nor agree | 47 | 16.5% |
|  | Somewhat agree | 13 | 4.6% |
|  | Completely agree | 10 | 3.5% |
| **Importance of STI Testing** | | **n** | **%** |
| To Stay Healthy | Not very important | 2 | 0.7% |
|  | Something important | 15 | 5.3% |
|  | Important | 61 | 21.5% |
|  | Very important | 206 | 72.5% |
| For peace of mind | Something important | 11 | 3.9% |
|  | Important | 49 | 17.3% |
|  | Very important | 224 | 78.9% |
| Not to feel ashamed | Not important | 26 | 9.2% |
|  | Not very important | 29 | 10.2% |
|  | Something important | 61 | 21.5% |
|  | Important | 70 | 24.6% |
|  | Very important | 98 | 34.5% |
| For information and advice | Not very important | 1 | 0.4% |
|  | Something important | 10 | 3.5% |
|  | Important | 57 | 20.1% |
|  | Very important | 216 | 76.1% |
| So I don't feel stressed | Not important | 8 | 2.8% |
|  | Not very important | 12 | 4.2% |
|  | Something important | 35 | 12.3% |
|  | Important | 63 | 22.2% |
|  | Very important | 166 | 58.5% |
| To feel comfortable | Not important | 4 | 1.4% |
|  | Not very important | 8 | 2.8% |
|  | Something important | 37 | 13.0% |
|  | Important | 69 | 24.3% |
|  | Very important | 166 | 58.5% |
| So as not to negatively affect me or future relationships | Not important | 2 | 0.7% |
|  | Not very important | 4 | 1.4% |
|  | Something important | 10 | 3.5% |
|  | Important | 47 | 16.5% |
|  | Very important | 221 | 77.8% |
| To be responsible | Something important | 7 | 2.5% |
|  | Important | 49 | 17.3% |
|  | Very important | 228 | 80.3% |
| To get the treatment I need before I have complications | Not important | 1 | 0.4% |
|  | Not very important | 2 | 0.7% |
|  | Something important | 7 | 2.5% |
|  | Important | 42 | 14.8% |
|  | Very important | 232 | 81.7% |
| So as not to negatively affect my future and career prospects | Not important | 7 | 2.5% |
|  | Not very important | 6 | 2.1% |
|  | Something important | 27 | 9.5% |
|  | Important | 52 | 18.3% |
|  | Very important | 192 | 67.6% |

**Supplementary Table 5.** Social fear of STI testing for medical students.

| **If I had an STI:** |  | **n** | **%** |
| --- | --- | --- | --- |
| I'd be embarrassed | Strongly disagree | 32 | 8.0% |
|  | Somewhat disagree | 36 | 9.2% |
|  | Neither disagree, nor agree | 68 | 19.3% |
|  | Somewhat agree | 101 | 30.2% |
|  | Completely agree | 47 | 12.6% |
| I'd be baffled | Strongly disagree | 19 | 4.2% |
|  | Somewhat disagree | 17 | 3.7% |
|  | Neither disagree, nor agree | 26 | 6.2% |
|  | Somewhat agree | 104 | 31.2% |
|  | Completely agree | 118 | 35.9% |
| People would avoid me | Strongly disagree | 29 | 7.1% |
|  | Somewhat disagree | 44 | 11.6% |
|  | Neither disagree, nor agree | 120 | 36.6% |
|  | Somewhat agree | 62 | 17.3% |
|  | Completely agree | 29 | 7.1% |
| People would think badly of me | Strongly disagree | 22 | 5.1% |
|  | Somewhat disagree | 28 | 6.8% |
|  | Neither disagree, nor agree | 83 | 24.2% |
|  | Somewhat agree | 107 | 32.2% |
|  | Completely agree | 44 | 11.6% |
| I'd be worried about my parents' reaction | Strongly disagree | 25 | 5.9% |
|  | Somewhat disagree | 21 | 4.8% |
|  | Neither disagree, nor agree | 26 | 6.2% |
|  | Somewhat agree | 82 | 23.8% |
|  | Completely agree | 130 | 40.0% |
| I'd be worried about my sexual partner's reaction | Strongly disagree | 14 | 2.9% |
|  | Somewhat disagree | 9 | 1.6% |
|  | Neither disagree, nor agree | 19 | 4.2% |
|  | Somewhat agree | 82 | 23.8% |
|  | Completely agree | 160 | 50.5% |
| I'd be worried about the lab staff gossiping about me | Strongly disagree | 130 | 40.0% |
|  | Somewhat disagree | 36 | 9.2% |
|  | Neither disagree, nor agree | 52 | 14.1% |
|  | Somewhat agree | 28 | 6.8% |
|  | Completely agree | 38 | 9.8% |
| I'd feel judged | Strongly disagree | 45 | 12.0% |
|  | Somewhat disagree | 41 | 10.7% |
|  | Neither disagree, nor agree | 65 | 18.3% |
|  | Somewhat agree | 71 | 20.2% |
|  | Completely agree | 62 | 17.3% |

**Supplementary Table 6.** Self-efficacy question toward STI testing in medical students.

| **How safe you are about getting tested for STIs if:** |  | **n** | **%** |
| --- | --- | --- | --- |
| The test site is a long way away | 0% Security | 18 | 6.3% |
|  | 25% security | 42 | 14.8% |
|  | 50% security | 68 | 23.9% |
|  | 75% security | 65 | 22.9% |
|  | 100% security | 91 | 32.0% |
| You have to go on your own | 0% Security | 3 | 1.1% |
|  | 25% security | 22 | 7.7% |
|  | 50% security | 49 | 17.3% |
|  | 75% security | 47 | 16.5% |
|  | 100% security | 163 | 57.4% |
| Addresses accompanied by other people | 0% Security | 11 | 3.9% |
|  | 25% security | 39 | 13.7% |
|  | 50% security | 69 | 24.3% |
|  | 75% security | 58 | 20.4% |
|  | 100% security | 107 | 37.7% |
| Had to wait for an appointment | 0% Security | 14 | 4.9% |
|  | 25% security | 30 | 10.6% |
|  | 50% security | 54 | 19.0% |
|  | 75% security | 66 | 23.2% |
|  | 100% security | 120 | 42.3% |
| If there is someone you know there (Taking the test/In the waiting room) | 0% Security | 23 | 8.1% |
|  | 25% security | 51 | 18.0% |
|  | 50% security | 71 | 25.0% |
|  | 75% security | 51 | 18.0% |
|  | 100% security | 88 | 31.0% |
| You meet someone you know at the site of the scans | 0% Security | 24 | 8.5% |
|  | 25% security | 60 | 21.1% |
|  | 50% security | 58 | 20.4% |
|  | 75% security | 60 | 21.1% |
|  | 100% security | 82 | 28.9% |
| He thinks he's going to be the biggest one in the waiting room | 0% Security | 59 | 20.8% |
|  | 25% security | 53 | 18.7% |
|  | 50% security | 48 | 16.9% |
|  | 75% security | 34 | 12.0% |
|  | 100% security | 90 | 31.7% |
| You don't have time available | 0% Security | 42 | 14.8% |
|  | 25% security | 60 | 21.1% |
|  | 50% security | 71 | 25.0% |
|  | 75% security | 46 | 16.2% |
|  | 100% security | 65 | 22.9% |
| Not sure if the symptom is not a symptom of an STI | 0% Security | 17 | 6.0% |
|  | 25% security | 39 | 13.7% |
|  | 50% security | 74 | 26.1% |
|  | 75% security | 45 | 15.8% |
|  | 100% security | 109 | 38.4% |
| My friends have had bad experiences | 0% Security | 25 | 8.8% |
|  | 25% security | 49 | 17.3% |
|  | 50% security | 79 | 27.8% |
|  | 75% security | 47 | 16.5% |
|  | 100% security | 84 | 29.6% |
| You've had bad experiences in the past | 0% Security | 18 | 6.3% |
|  | 25% security | 49 | 17.3% |
|  | 50% security | 75 | 26.4% |
|  | 75% security | 49 | 17.3% |
|  | 100% security | 93 | 32.7% |
| Appointments became available over the weekend | 0% Security | 8 | 2.8% |
|  | 25% security | 27 | 9.5% |
|  | 50% security | 51 | 18.0% |
|  | 75% security | 51 | 18.0% |
|  | 100% security | 147 | 51.8% |
| Appointments were available in the afternoon | 0% Security | 7 | 2.5% |
|  | 25% security | 25 | 8.8% |
|  | 50% security | 53 | 18.7% |
|  | 75% security | 52 | 18.3% |
|  | 100% security | 147 | 51.8% |
| The result of the test was published | 0% Security | 120 | 42.3% |
|  | 25% security | 55 | 19.4% |
|  | 50% security | 45 | 15.8% |
|  | 75% security | 24 | 8.5% |
|  | 100% security | 40 | 14.1% |
| You may be able to complete the test at home | 0% Security | 34 | 12.0% |
|  | 25% security | 31 | 10.9% |
|  | 50% security | 51 | 18.0% |
|  | 75% security | 42 | 14.8% |
|  | 100% security | 126 | 44.4% |
| Appointments were available in the morning | 0% Security | 10 | 3.5% |
|  | 25% security | 29 | 10.2% |
|  | 50% security | 61 | 21.5% |
|  | 75% security | 50 | 17.6% |
|  | 100% security | 134 | 47.2% |
| Appointments were available in the evening | 0% Security | 39 | 13.7% |
|  | 25% security | 26 | 9.2% |
|  | 50% security | 55 | 19.4% |
|  | 75% security | 40 | 14.1% |
|  | 100% security | 124 | 43.7% |
