# Supplementary material for: Exploring psychosocial factors influencing sexually transmitted infection intention testing among medical students: a cross-sectional study in two universities
Source: Front Public Health. 2024 Sep 20;12:1407070. doi: 10.3389/fpubh.2024.1407070 (PMC11451435; doi:10.3389/fpubh.2024.1407070)
Supplement: Supplementary file 2 [file Table_2.DOCX]

# **SURVEY**

**SECTION 1.** SOCIODEMOGRAPHIC CHARACTERIZATION, SELF-REPORTED SEXUAL BEHAVIOR AND PERFORMANCE OF PARACLINICS IN THE FACE OF STIS.

- Biological Sex

Female

Male

- How old are you?

------------------------------

- Which university you are studying at

Autonomous University Foundation of the Americas

Technological University of Pereira

- What semester are you currently studying?

I (First)

II (Second)

III (Third)

IV (Fourth)

V (Fifth)

VI (Sixth)

- What is your religious stance OR BELIEF?

Catholic

Christian

Protestant

Agnostic

Atheist

Non-believer

Other

Have you ever had a sex relationship (at least one time prior this survey)?

Yes

No

| Sexual intercourse includes all of the following: |
| --- |
| •Vaginal sex (penis-in-vagina intercourse)  • Oral sex (mouth-to-genital contact)  • Anal sex (penis-in-anus)  • Genital hand contact |
|  |
|  |
|  |

- Have you ever been tested for an STI?

If in the last 6 months

Yes, but not in the last 6 months

No

| What is an STI? |
| --- |
| STIs (also known as sexually transmitted infections, or STDs for 'sexually transmitted diseases', or EVs for 'venereal diseases') are infections that have a high chance of being spread from person to person through unprotected sex. Examples are syphilis, gonorrhea, chlamydia, trichomoniasis, hepatitis B, herpes, HIV, and HPV. |
|  |
|  |
|  |
|  |
|  |

Please give an answer to each question below

| Have you ever had... | | |
| --- | --- | --- |
| Vaginal intercourse with a stable partner without a condom? | Yes | No |
| Vaginal intercourse with an occasional partner without a condom? | Yes | No |
| Anal intercourse with a stable partner without a condom? | Yes | No |
| Anal intercourse with a casual partner without a condom? | Yes | No |

| What is a stable couple? |
| --- |
| Someone with whom you have a sexual relationship and interaction, e.g., boyfriend/girlfriend, spouse |
|  |
| What is a casual partner? |
| Someone with whom you have sex on one or more occasions, but you are not in a relationship. |
|  |

**SECTION 2:** Psychosocial Factors

KNOWLEDGE

Please indicate whether you believe each statement below is TRUE (V), FALSE (F), I DON'T KNOW.

**If you don't know, don't guess, choose don't know.

|  | V/Si | F/No | I do not know |
| --- | --- | --- | --- |
| 1. You can always tell if you have an STI, as you would have symptoms (e.g., changes in your body) |  |  |  |
|  |  |  |  |
|  |  |  |  |
| 1. The symptoms of all sexually transmitted diseases are painful |  |  |  |
|  |  |  |  |
| 1. If you only have unprotected sex once, you won't get infected with an STI |  |  |  |
|  |  |  |  |
| 1. People who use condoms are always safe from all STIs |  |  |  |
|  |  |  |  |
| 1. Some STIs can cause infertility in women and men |  |  |  |
|  |  |  |  |
| 1. Not All Sexually Transmitted Infections Can Be Cured |  |  |  |
|  |  |  |  |
| 1. Most STIs will go away on their own |  |  |  |
| 1. Some STIs can be treated with antibiotics |  |  |  |
| 1. You have to pay to get tested |  |  |  |
| 1. If you don't have symptoms (but want a test), the best place to get tested is at your Health Care Provider (EPS) |  |  |  |
|  |  |  |  |
|  |  |  |  |
|  |  |  |  |
| 1. Are aware of the costs of getting tested for STIs |  |  |  |
| 1. Know where to get tested for STIs |  |  |  |
| 1. Requires a medical prescription to be tested for one or more STIs |  |  |  |

Which of the following can be diagnosed by taking a sample of your urine?

**If you don't know, don't guess, choose don't know.

|  | Yes | No | I do not know |
| --- | --- | --- | --- |
| Chlamydia |  |  |  |
| Gonorrhea |  |  |  |
| HIV |  |  |  |
| Syphilis |  |  |  |

### SOCIAL PRESSURE

Please use the scale below to respond to the following statements.

**This is a numerical scale ranging from 1 to 5, with 1 being ***the*** equivalent of ***strongly disagree*** and ***5*** being the equivalent ***of strongly agree.***

|  |  |  |  |  | 1 | 2 | 3 | 4 | 5 |
| --- | --- | --- | --- | --- | --- | --- | --- | --- | --- |
| My sexual partners would like to be tested for STIs | | | | |  |  |  |  |  |
|  |  |  |  |  |  |  |  |  |  |
| My friends would want to be tested for STIs | | | | |  |  |  |  |  |
|  |  |  |  |  |  |  |  |  |  |
| My family would like to be tested for STIs | | | | |  |  |  |  |  |
|  |  |  |  |  |  |  |  |  |  |
| Doctors and health care professionals would want me tested for STIs | | | | |  |  |  |  |  |
|  |  |  |  |  |  |  |  |  |  |

| Sexual Partners |
| --- |
| These include stable or casual partners with whom you have had sex |
|  |

Please use the scale below to respond to the following statements

**This is a numerical scale that goes from 1 to 5, with 1 being ***the*** equivalent of ***completely disagreeing to take an STI test*** and ***5*** being completely ***in agreement to take an STI test.***

|  |  |  |  |  | 1 | 2 | 3 | 4 | 5 |
| --- | --- | --- | --- | --- | --- | --- | --- | --- | --- |
| If I knew my sexual partners wanted me to get tested for STIs, I would... | | | | |  |  |  |  |  |
|  |  |  |  |  |  |  |  |  |  |
| If I knew my friends wanted me to get tested for STIs, I would... | | | | |  |  |  |  |  |
|  |  |  |  |  |  |  |  |  |  |
| If I knew my family wanted me to get tested for STIs, I would... | | | | |  |  |  |  |  |
|  |  |  |  |  |  |  |  |  |  |
| If I knew my doctors and health care professionals wanted me to get tested for STIs, I would... | | | | |  |  |  |  |  |
|  |  |  |  |  |  |  |  |  |  |

### ATTITUDES

***Direct***

*Please use the scale below to respond to the statement.* It is a numerical scale ranging from 1 to 5, with ***1*** being the equivalent of ***completely disagreeing*** and ***5*** being ***the equivalent of completely agreeing.***

***Getting tested for STIs is...***

| A Waste of Time | |  |  | |
| --- | --- | --- | --- | --- |
| 1 | 2 | 3 | 4 | 5 |
| Something good for me | |  |  | |
| 1 | 2 | 3 | 4 | 5 |
| Something Painful | |  |  | |
| 1 | 2 | 3 | 4 | 5 |
| Somewhat boring | |  |  | |
| 1 | 2 | 3 | 4 | 5 |
| Somewhat reckless | |  |  | |
| 1 | 2 | 3 | 4 | 5 |
| Something Harmful | |  |  | |
| 1 | 2 | 3 | 4 | 5 |
| Something Unattractive | |  |  | |
| 1 | 2 | 3 | 4 | 5 |
| Something Unimportant | |  |  | |
| 1 | 2 | 3 | 4 | 5 |

***Indirect***

Use the following scales to respond to the statement.

"If I go to STI testing:"

**This is a numerical scale ranging from 1 to 5, with ***1 being the*** equivalent of ***Strongly Disagree*** and ***5*** being the equivalent ***of Strongly Agree***

|  | 1 | 2 | 3 | 4 | 5 |
| --- | --- | --- | --- | --- | --- |
| I'm going to stay healthy |  |  |  |  |  |
| I feel calm |  |  |  |  |  |
| It will be embarrassing |  |  |  |  |  |
| I'm going to get information and advice |  |  |  |  |  |
| It will be stressful |  |  |  |  |  |
| I'm going to get comfortable |  |  |  |  |  |
| That will negatively affect my future and my relationships |  |  |  |  |  |
|  |  |  |  |  |  |
| I'll feel responsible |  |  |  |  |  |
| It will take a long time |  |  |  |  |  |
| I will get the treatment I need before I have complications. |  |  |  |  |  |
|  |  |  |  |  |  |
| It will negatively affect my future and career prospects |  |  |  |  |  |
|  |  |  |  |  |  |

"Outcome Evaluations"

Use the scales to respond to each statement.

**This is a numerical scale ranging from 1 to 5, with 1 being ***the*** equivalent of ***Not Important*** and ***5*** being  ***the equivalent of Very Important***

|  | 1 | 2 | 3 | 4 | 5 |
| --- | --- | --- | --- | --- | --- |
| To stay healthy is |  |  |  |  |  |
| To get peace of mind is |  |  |  |  |  |
| Not to feel ashamed is |  |  |  |  |  |
| For information and advice is |  |  |  |  |  |
| To avoid feeling stressed, it's |  |  |  |  |  |
| To feel comfortable is |  |  |  |  |  |
| In order not to negatively affect me or future relationships, it is |  |  |  |  |  |
|  |  |  |  |  |  |
| To be responsible |  |  |  |  |  |
| To get the treatment I need before I have complications. |  |  |  |  |  |
|  |  |  |  |  |  |
| So as not to negatively affect my future and career prospects |  |  |  |  |  |
|  |  |  |  |  |  |

SOCIAL FEAR

Use the scales to respond to the statement.

If I had an STI...

**This is a numerical scale ranging from 1 to 5, with ***1 being the*** equivalent of ***Strongly Disagree*** and ***5*** being the equivalent ***of Strongly Agree***

|  | 1 | 2 | 3 | 4 | 5 |
| --- | --- | --- | --- | --- | --- |
| I'd be ashamed |  |  |  |  |  |
| I'd be baffled |  |  |  |  |  |
| People would avoid me |  |  |  |  |  |
| People would think badly of me |  |  |  |  |  |
| I'd be worried about my parents' reaction |  |  |  |  |  |
|  |  |  |  |  |  |
| I'd be worried about my sexual partner's reaction |  |  |  |  |  |
|  |  |  |  |  |  |
| I'd be worried about the lab staff gossiping about me |  |  |  |  |  |
|  |  |  |  |  |  |
| I'd feel judged |  |  |  |  |  |

EFFICACY

Use the scales to respond to the statement.

**How confident you are about getting tested for STIs if...**

|  | 0% | 25% | 50% | 75% | 100% |
| --- | --- | --- | --- | --- | --- |
| If the test site is too far away |  |  |  |  |  |
| If you have to go on your own |  |  |  |  |  |
| If you are addressing with other people |  |  |  |  |  |
| If I had to wait for an appointment |  |  |  |  |  |
| If you know someone there (Taking the Test/Waiting Room) |  |  |  |  |  |
|  |  |  |  |  |  |
| If you find someone you know |  |  |  |  |  |
| He thinks he's going to be older than everyone else in the waiting room |  |  |  |  |  |
|  |  |  |  |  |  |
| If you don't have time |  |  |  |  |  |
| I'm not sure if the symptom isn't a symptom of an STI |  |  |  |  |  |
|  |  |  |  |  |  |
| If my friends have had bad experiences |  |  |  |  |  |
|  |  |  |  |  |  |
| If you have had bad experiences in the past |  |  |  |  |  |
|  |  |  |  |  |  |
| If appointments were available on the weekend |  |  |  |  |  |
|  |  |  |  |  |  |
| If appointments were available in the morning |  |  |  |  |  |
|  |  |  |  |  |  |
| If the proof were published |  |  |  |  |  |
|  |  |  |  |  |  |
| If I could complete the test at home |  |  |  |  |  |
| If appointments were available in the afternoon |  |  |  |  |  |
| If appointments were available in the evening |  |  |  |  |  |

INTENTION

Answer the following question:

Do I intend to get tested for STIs in the next month?

- Yes
- No
